# Tau deposition patterns within the medial temporal lobe follow the connectivity of the entorhinal and perirhinal cortex – a combined post-mortem and 7T fMRI study

**DOI:** 10.21203/rs.3.rs-10581799/v1

**Published:** 2026-09-02

**Authors:** Hannah de Bruin, Xenia Przygodda, Laura E. M. Wisse, Sadhana Ravikumar, Emrah Duezel, Yolande A. L. Pijnenburg, Paul A. Yushkevich, Colin Groot, David Berron, Nicolai Franzmeier, Rik Ossenkoppele

**Affiliations:** Vrije Universiteit Amsterdam, Amsterdam UMC location VUmc; Otto-von-Guericke University; Lund University; University of Pennsylvania; Otto-von-Guericke University; Vrije Universiteit Amsterdam, Amsterdam UMC location VUmc; University of Pennsylvania; Vrije Universiteit Amsterdam, Amsterdam UMC location VUmc; German Center for Neurodegenerative Diseases (DZNE); University Hospital, Ludwig Maximilian University of Munich; Vrije Universiteit Amsterdam, Amsterdam UMC location VUmc

**Keywords:** Alzheimer’s disease, medial temporal lobe, post-mortem, AT8 tau, 7 tesla functional MRI, functional connectivity

## Abstract

In aging, tau pathology can develop in the medial temporal lobe (MTL), but generally only progresses to the cortex in the context of amyloidosis, driving neurodegeneration and Alzheimer’s disease (AD) dementia. Functional connectivity shapes this cortical tau expansion, supporting trans-neuronal tau spreading. However, it is unclear whether MTL tau accumulation itself follows within-MTL connections – particularly those of the transentorhinal (Brodmann area 35 [A35]) and entorhinal cortex (ErC), the earliest cortical sites of tau accumulation in AD and variably affected in aging. To address this, we assessed three-dimensional neurofibrillary tangle (NFT) burden across 14 MTL subregions using post-mortem quantitative histopathology in 25 individuals (*n* = 4 amyloid-β negative; *n* = 21 amyloid-β positive) on the AD continuum. These data were combined with high-resolution 7T resting-state functional MRI-based functional connectivity among the same 14 subregions obtained from 30 young healthy controls. We examined covariance in NFT burden across MTL subregions and tested associations with functional connectivity strength. We then applied seed-based connectivity analyses to identify which MTL subregion’s connectivity profile best predicted MTL NFT patterns. NFT levels were highest and comparable across A35 (0.62 ± 0.40), posterior cornu ammonis 1 (CA1; 0.61 ± 0.46), anterolateral ErC (alErC; 0.60 ± 0.39), and anterior CA1 (0.57 ± 0.43). We found a significant positive association between MTL connectivity and covariance in MTL NFTs (*β* = 0.49, *p* < 0.001), with closely connected MTL regions showing similar NFT levels. When assessing seed-based connectivity as a predictor of NFT patterns, alErC (*ρ* = 0.66, *p* < 0.001), A36 (*ρ* = 0.64, *p* < 0.001), and posteromedial ErC (pmErC; *ρ* = 0.59, *p* < 0.001) connectivity showed the strongest, comparable correspondence with MTL NFT deposition. This pattern was consistent within low (Braak 0–II) and high (Braak III–VI) NFT burden groups. These results suggest that within-MTL tau propagation is shaped by intrinsic network organization, highlighting entorhinal and perirhinal connectivity profiles as potential drivers of MTL tau progression and as candidate targets for therapeutic interventions.

## Introduction

Alzheimer’s disease (AD) is characterized by the accumulation of extracellular amyloid-β plaques and intracellular neurofibrillary tangles (NFTs) composed of hyperphosphorylated tau, with tau showing the strongest association with neurodegeneration and cognitive decline [1–3]. Although tau pathology is often detected in the medial temporal lobe (MTL) in ageing [4–6], its progression beyond the MTL into the neocortex depends on the presence of amyloid-β [7–11]. This MTL-confined stage may represent a critical window, during which the spatial pattern of tau accumulation forms the substrate from which subsequent cortical spreading – once amyloid-β is present – will originate. This highlights the importance of understanding how tau propagates within the MTL.

It is established that tau progression does not occur randomly, but instead follows the brain’s intrinsic network architecture [12–21]. Studies using tau positron emission tomography (PET) have shown that functional connectivity can predict the spatial distribution and progression of tau deposition, such that tau appears to propagate from regions affected early in the disease to regions that are functionally strongly connected to those regions [16–21]. These findings are consistent with the hypothesis of trans-neuronal tau propagation [22–28]. In line with this framework, functional connectivity has also been shown to predict the progression of tau from the MTL to the neocortex [12, 29], suggesting that large-scale brain networks may shape the progression of tau pathology.

However, it remains unclear whether a similar connectivity-based mechanism is involved in tau progression within the MTL itself. This is a key open question, as the MTL includes the earliest cortical regions affected by tau pathology and plays a central role in the initial stages of AD. According to the Braak staging framework, the transentorhinal region, broadly corresponding to Brodmann area 35 (area 35 perirhinal cortex; A35, see Fig. 1), is the first cortical region in which tau pathology originates [1, 30–33]. It then progresses to the entorhinal cortex (ErC), first affecting the anterolateral entorhinal cortex (alErC) and subsequently the posteromedial entorhinal cortex (pmErC) [1, 30–33]. From there, tau pathology progresses into the hippocampal formation, initially involving cornu ammonis 1 (CA1), followed by the subiculum [1, 30–33]. Tau pathology then extends to Brodmann area 36 (area 36 perirhinal cortex; A36), before later involving cornu ammonis 3 (CA3) and the dentate gyrus (DG) [1, 30–33]. If tau indeed propagates trans-neuronally, tau accumulation within the MTL would be expected to follow the intrinsic connections of the earliest affected regions, particularly A35 and the ErC.

These post-mortem observations have been supported by *in vivo* tau-PET studies [29, 34]. However, investigating within-MTL tau and connectivity associations *in vivo* is challenging because tau is difficult to quantify at the level of the individual MTL subregions, due to the limited spatial resolution and off-target binding of tau-PET [29, 34–39]. In addition, these subregions are small, show inter-individual variability in shape and size, and can thus be challenging to delineate reliably using clinic-grade magnetic resonance imaging (MRI), as gold-standard cytoarchitectonic borders are not visible – although more robust MTL subfield delineation is feasible at higher magnetic field strengths [40–43]. Post-mortem histopathology mitigates the first limitation by enabling direct, high-resolution assessment of regional NFT burden in the human brain, whereas ultra-high-field 7 tesla (7T) MRI addresses the second limitation by providing the anatomical precision needed to study MTL subregions and compute their functional connectivity profiles [33, 42–44]. Combined, these approaches provide a unique opportunity to investigate whether local patterns of NFT burden reflect the intrinsic connectivity architecture of the MTL.

Therefore, we examined whether patterns of post-mortem MTL NFT burden across 14 subregions in 25 (*n* = 4 amyloid-β negative; *n* = 21 amyloid-β positive) individuals with varying NFT levels are associated with within-MTL normative functional connectivity strength. Connectivity was assessed using high-resolution 7T functional MRI (fMRI) from a separate cohort of 30 young healthy controls. Specifically, we aimed to (i) confirm that the distribution of NFT burden across MTL subregions is consistent with prior findings from the same dataset using a slightly different segmentation protocol [33], and investigate (ii) whether covariance in NFT deposition across MTL subregions is associated with within-MTL functional connectivity, and (iii) which MTL seed region’s connectivity profile best predicts the spatial distribution of NFT burden across the MTL. We hypothesize that NFT burden will be highest in A35, that more strongly functionally connected MTL regions will show greater covariance in NFT burden, and that the functional connectivity profile of the earliest affected regions – particularly A35 and the ErC – will best predict the distribution of NFT pathology within the MTL.

## Materials and methods

### Donor cohort

We obtained autopsy data from the OpenNeuro repository (https://openneuro.org/datasets/ds004767/versions/1.0.0), restricting to brain donors on the AD continuum for whom NFT burden maps were available [33]. This yielded a sample of 25 donors, of whom *n* = 9 from the University of Pennsylvania (UPENN) and *n* = 16 from the Human Neuroanatomy Laboratory at the University of Castilla La Mancha (UCLM). UPENN donors were drawn from *in vivo* research cohorts on ageing and dementia and included patients at the Penn Alzheimer’s Disease Core Center and the Penn Frontotemporal Degeneration Center. UCLM donors were derived from the general population and were primarily older individuals without known neurological disorders. Human brain samples were obtained in accordance with local laws and regulations and approved by local medical ethical committees. Where possible, pre-consent during life and, in all cases, next-of-kin consent at death was obtained.

### MRI acquisition

Post-mortem brain tissue samples underwent high-resolution *ex vivo* MRI using a previously described pipeline [33, 44]. First, after fixation, the MTL was dissected from one brain hemisphere (the selected hemisphere varied across cases) and scanned using a 7T human MRI scanner with 0.4 mm isotropic resolution and a 9.4T animal MRI scanner with 0.2 mm isotropic resolution (both T2-weighted). Following image acquisition, 9.4T images were corrected for bias field non-uniformity and normalized to a common intensity range. Geometric distortions in the 9.4T MRI were corrected using a phantom-based linear scaling procedure [33]. The 7T scan was used to generate a specimen-specific three-dimensional (3D)-printed mould to guide tissue sectioning orthogonal to the longitudinal axis of the hippocampus, facilitating histology-MRI alignment.

### Histological processing and tau staining

Following MRI acquisition, the MTL specimens were sectioned into 20 mm thick tissue blocks using the aforementioned 3D-printed custom mould for each specimen and subsequently cryosectioned into serial 50-μm sections without gaps. During sectioning, block-face photographs were acquired to aid 3D reconstruction and registration. Every 10th section was stained for Nissl, while every 20th adjacent section was stained for phosphorylated tau (AT8) and counterstained with Nissl. The stained sections were mounted on glass slides, digitized at pixel size 0.4 μm at 20x magnification, and uploaded to a digital histology platform for visualization, anatomical labelling, and further analysis. Full details of tissue preparation and staining procedures are described elsewhere [33, 44].

### Derivation of 2D NFT burden maps

To quantify NFT burden on anti-tau histology sections, a weakly supervised learning (WSL) approach was applied [33, 44]. First, raters manually labelled small regions (patches) from digitized slides as showing either tangle or non-tangle tissue. These labelled image regions were then used to train the WSL WildCat model on patches sampled across slides, which was subsequently applied to the full set of anti-tau slides to generate spatial heat maps reflecting the location and relative intensity of NFT pathology, resulting in whole-slide 2D NFT burden maps representing regional NFT burden.

### Histology-to-MRI reconstruction

To reconstruct histology in MRI space, a multi-stage registration pipeline was applied [33, 44]. Block-face images were first stacked in 3D and aligned to the 9.4T MRI, followed by registration of Nissl sections to MRI and anti-tau sections to the corresponding Nissl sections. The resulting transformations were combined to map histology-derived NFT burden maps into MRI space, yielding 3D reconstructions of tau pathology for each specimen. Individual MRI and NFT maps were subsequently co-registered in a common template space using the registration pipeline described in Ravikumar et al. [33], enabling regional analyses of NFT burden across anatomical regions of interest (ROIs). Average NFT burden across specimens was computed and visualized in template space.

### Regions of interest for assessment of NFT burden

Regional NFT burden was assessed in MTL cortical subregions and hippocampal subfields. The included regions were ErC, A35, A36, PHC, CA1, subiculum, CA3, DG, and the hippocampal tail (Fig. 1). CA2 was not included as a separate ROI, hence the gap between CA1 and CA3 in the figure. The resolution of the *ex vivo* MRI template allowed landmark-based manual labelling of the MTL by a highly experienced anatomist [45, 46]. To match ROIs for functional connectivity as closely as possible, anatomical labels were manually adjusted. CA1, subiculum, and CA3 borders were adjusted according to the Berron protocol [43]. ErC division into anterolateral and posteromedial subregions was approached by merging subregions Eo (olfactory), Er (rostral), ELr (lateral rostral), EMI (medial intermediate), and ELc (lateral caudal) into alErC, and subregions EI (intermediate), EC (caudal), and ECL (caudal limiting) into pmErC [46, 47]. The PHC ROI was obtained by merging area TF, area TH, and the temporopolar cortex [45, 46]. CA1, subiculum, CA3, and DG were divided into anterior and posterior subregions according to the tau data. For each region in each specimen, NFT burden was summarized using the mean of voxel values within the region, considering only voxels for which histology-derived measures were available.

### Functional connectivity

Comparable MTL ROIs were used to assess ROI-to-ROI functional connectivity, manually segmented according to the Berron protocol [43] and using an ErC-specific delineation [47]. Bilateral functional connectivity measures were obtained from a separate cohort of 30 young healthy controls (16 female, mean age 25.7 years) who underwent high-resolution 7T resting-state fMRI at 1×1×1 mm isotropic voxel size, enabling characterization of functional connectivity patterns among MTL subregions. ROI-to-ROI functional connectivity was computed as Fisher z-transformed bivariate Pearson correlations between fMRI time series (i.e., fluctuations in the blood oxygen level-dependent [BOLD] signal) of all ROI pairs. We used the average of left-hemispheric and right-hemispheric functional connectivity for our analyses. Full details on scan protocol and preprocessing are reported elsewhere [48, 49].

### Statistical analyses

Mean NFT load was computed per subject for each of the 14 MTL regions. Regional differences in NFT load were assessed using pairwise within-subject (paired-samples) *t*-tests comparing regions, with false discovery rate (FDR) correction applied across all region pairs.

For each pair of MTL ROIs, NFT covariance was calculated as the partial Pearson correlation between post-mortem quantitative NFT ratings across participants, adjusted for age and sex. The resulting correlation coefficients were Fisher z-transformed. The association between functional connectivity and NFT covariance was examined using linear regression, with age- and sex-adjusted NFT covariance as the outcome and functional connectivity as the predictor. Statistical significance was assessed by comparing the observed standardized regression coefficient against a null distribution generated by randomly rewiring the functional connectivity network 1000 times, preserving the degree sequence and overall edge-weight distribution; the resulting *p*-value reflects the proportion of null-model coefficients equal to or exceeding the observed coefficient.

To identify which MTL region most closely resembled a tau progression seed, a seed-based connectivity approach was used. For each ROI serving as a seed region, the functional connectivity profile of that seed (i.e., its group-level functional connectivity with all non-seed regions) was correlated with the group-average NFT load in the remaining ROIs using Spearman rank correlations. A bootstrapping procedure (*n* = 1000 iterations, with replacement) was used to resample subjects, recompute the group-average NFT profile for each bootstrap sample, and recalculate the seed-specific Spearman correlation, yielding stable estimates of the mean seed-specific correlation with 95% confidence intervals. For each seed, statistical significance of the correlation was assessed by computing, across bootstrap iterations, the proportion of correlations falling on either side of zero and doubling the smaller proportion, with FDR correction applied across all seed regions. Seed regions were ranked by mean bootstrapped correlation. To test whether seed region correlations differed significantly from one another, pairwise bootstrap-based tests were conducted for all seed pairs. Specifically, for each bootstrap iteration, the difference between the bootstrapped seed-specific correlations of each seed pair was computed, and a two-sided *p*-value was derived by computing, across bootstrap iterations, the proportion of differences falling on either side of zero and doubling the smaller proportion. FDR correction was applied across all pairwise comparisons.

To validate the seed connectivity rankings in the context of known tau staging, post-mortem NFT data were stratified by Braak stage into a low-burden group (Braak 0–II) and a high-burden group (Braak III–VI) [33]. The seed-based bootstrapping procedure described above was applied separately within each group, yielding group-specific estimates of the mean bootstrapped Spearman correlation per seed ROI with 95% confidence intervals. Pairwise bootstrap-based comparisons were conducted within each group, with FDR correction applied across all pairwise comparisons per group.

All analyses were conducted in RStudio (version 4.3.2). Statistical significance was set at *p* < 0.05 (FDRcorrected, where applicable). ITK-SNAP [50] (version 4.4.0) and 3D Slicer (version 5.10.0) were used for visualizations of MTL regions.

## Results

### Sample characteristics

We included 25 donors on the AD continuum with varying NFT levels, selected based on the availability of NFT burden maps [33]. An overview of the sample characteristics can be found in Table 1. Mean age at death was 78.60 ± 10.95 years, and the sample included more males (64%) than females (36%). According to the CERAD ABC-criteria for AD neuropathologic change (ADNC) [51], a substantial subset of cases (40%) had widespread amyloid-β pathology (A3 score). With regard to NFTs, 4% had no detectable NFT burden (B0 score), 40% had low NFT burden (B1 score; Braak stage I or II), 40% had intermediate NFT burden (B2 score; Braak stage III or IV), and 16% had high NFT burden (B3 score; Braak stage V or VI). There was variability in neuritic plaque burden (C scores varied from 0 to 3) across cases. ADNC was the predominant neuropathological diagnosis (32% low, 24% intermediate, 12% high), although other primary neuropathological diagnoses were also identified (i.e., Lewy body disease, Primary Age-Related Tauopathy [PART], or cerebrovascular disease). Additional neuropathological diagnoses (e.g., cerebral amyloid angiopathy [CAA], limbic-predominant age-related TDP-43 encephalopathy [LATE], and corticobasal degeneration [CBD]) were identified in a subset of cases, but only as secondary pathology.

### Mean post-mortem NFT load across MTL regions

Prior to performing our connectivity analyses, we aimed to confirm that the distribution of NFT burden across MTL subregions was consistent with the original analysis using a slightly different segmentation protocol [33]. The mean NFT load in all MTL regions across the 25 cases is summarized in Fig. 2A, accompanied by a visual representation of NFT burden in coronal slices (**B**). A35 demonstrated the highest overall NFT load (0.62 ± 0.40), differing significantly from the pmErC and A36 (*p* < 0.05), as well as from the anterior subiculum, PHC, hippocampal tail, posterior subiculum, posterior CA3, posterior DG, anterior CA3, and anterior DG (*p* < 0.001) (**Supplementary Table 1**). NFT burden of the posterior CA1 (0.61 ± 0.46), alErC (0.60 ± 0.39), and anterior CA1 (0.57 ± 0.43) were statistically comparable to that of A35. These findings are largely consistent with the original analysis [33].

### Association between functional connectivity and post-mortem NFT covariance

Our second aim was to examine whether patterns of NFT deposition are associated with within-MTL functional connectivity. Figure 3A visualizes pairwise functional connectivity metrics across MTL regions from 30 young healthy controls, and Fig. 3B displays age- and sex adjusted MTL NFT covariance metrics of the 25 post-mortem cases included in this study. When statistically testing the association between these two (Fig. 3C, **scatterplot**), we found a significant positive association (*β* = 0.49). To assess the significance of this association beyond what would be expected by chance, we compared the observed standardized regression coefficient against a null distribution generated by randomly rewiring the functional connectivity network 1000 times while preserving its degree sequence and overall edge-weight distribution (Fig. 3C, **beeswarm plot**). We found that the observed *β* exceeded all null-model coefficients (*p* < 0.001), indicating that regions with strong functional connectivity showed greater similarity in NFT load than expected under a randomly organized network architecture.

### Association between seed ROI functional connectivity and post-mortem NFT load

The third aim of this study was to assess which MTL seed region best predicts the distribution of NFT burden across the MTL. For each of the 14 MTL subregions, we computed the seed-specific Spearman correlation between that region's functional connectivity profile and the group-average NFT load in the remaining ROIs, using a bootstrapping procedure (1000 iterations, resampling subjects with replacement) to obtain stable estimates with 95% confidence intervals. The strongest positive association was observed in alErC (*ρ* = 0.66, 95% CI [0.56, 0.78], *p* < 0.001), followed by A36 (*ρ* = 0.64, 95% CI [0.58, 0.71], *p* < 0.001) and pmErC (*ρ* = 0.59, 95% CI [0.51, 0.69], *p* < 0.001) (Fig. 4). Pairwise bootstrap-based comparisons with FDR correction showed that these three seed regions did not differ significantly from one another (**Supplementary Table 2**), indicating that the spatial pattern of NFTs across the MTL was comparably well explained by the functional connectivity profile of alErC, A36, and pmErC. These seed regions showed significantly stronger associations than most other MTL subregions, with the exception of pmErC versus A35 (*p* = 0.074; **Supplementary Table 2**). To further explore whether this pattern held across disease stages, we repeated the seed-based bootstrapping procedure separately within the low (i.e., Braak 0–II; *n* = 11) and high (i.e., Braak III–VI; *n* = 14) NFT burden groups. Notably, also within both the low and high Braak group, the alErC, A36, and pmErC emerged as the three MTL ROIs with the strongest seed-based associations (**Supplementary Figs. 1A and 2A**), although none differed significantly from A35 within either subgroup (**Supplementary Tables 3 and 4**). Visual representations of NFT burden in the low and high Braak groups can be found in **Supplementary Figs. 1B and 2B**, respectively. Interestingly, while alErC, A36, and pmErC were all the strongest predictors in both subgroups, the seed-based association for A36 was numerically higher in the high Braak group (*ρ* = 0.65, 95% CI [0.55, 0.73]) (**Supplementary Fig. 2**) than in the low Braak group (*ρ* = 0.60, 95% CI [0.41, 0.72]) (**Supplementary Fig. 1**), indicating that A36 connectivity might become more relevant as MTL tau pathology advances. Together, these findings suggest that entorhinal (alErC, pmErC) and perirhinal (A36) connectivity consistently shape MTL tau pathology patterns across disease stages.

## Discussion

The primary aim of this study was to examine whether patterns of post-mortem MTL NFT burden are associated with within-MTL functional connectivity. To address this, we combined post-mortem quantitative histopathology of 3D NFT burden across 14 MTL subregions with high-resolution 7T resting-state fMRI-based functional connectivity among those same subregions. NFT burden was assessed in 25 individuals (*n* = 4 amyloid-β negative, *n* = 21 amyloid-β positive) on the AD continuum with varying NFT levels, and functional connectivity was derived from 30 young healthy controls. In line with our expectations, NFT burden was highest in A35, alErC, and CA1. Importantly, regions with stronger functional connectivity showed greater covariance in NFT deposition, suggesting that intrinsic connectivity patterns shape tau distribution within the MTL. Moreover, functional connectivity profiles of ErC and A36 were the most robust predictors of MTL NFT deposition across disease stages. Together, these findings suggest that the propagation of tau within the MTL follows the network architecture of entorhinal and perirhinal regions, highlighting their connectivity as potential targets for early intervention to limit tau progression.

Consistent with established neuropathological staging and prior work using the same data but with a slightly different segmentation protocol, mean NFT load was highest in A35, alErC and CA1 – regions known to be among the earliest affected according to the Braak NFT staging scheme [1, 30, 31, 33]. Since regions affected earliest have had the longest time to accumulate tau pathology [17, 52, 53], high NFT burden is anticipated. Furthermore, our observed association between functional connectivity and NFT covariance is in line with previous (mainly) tau-PET studies demonstrating that tau covariance patterns closely mirror intrinsic functional connectivity networks [12, 13, 16, 20], and may be consistent with the transneuronal spreading hypothesis [22–28]. However, alternative explanations cannot be excluded, as regions sharing functional connectivity could also share transcriptomic profiles or other molecular vulnerabilities that independently promote tau accumulation [54]. Moreover, functional connectivity does not necessarily reflect (direct) structural connections between regions, meaning the anatomical substrate for potential transneuronal tau propagation remains uncertain [55]. Additionally, the cross-sectional nature of our data means that the directionality of tau progression cannot be directly inferred; the observed associations are consistent with network-based propagation but do not prove it. Nonetheless, our results add to the growing body of evidence that functional connectivity plays an important role in shaping tau deposition patterns – extending this concept from the neocortical level, where it has been well established using tau-PET imaging in large cohorts [12–21], to the fine-grained architecture of the MTL. Notably, prior studies have established that tau epicenters – regions with the highest tau burden – tend to show connectivity profiles that are closely aligned with the spatial distribution of tau pathology in the rest of the brain [16–21]. Our seed-based approach was designed to test this framework explicitly within the substructures of the MTL. We found that the connectivity profiles of ErC and A36 were the strongest overall predictors of MTL NFT distribution. We anticipated the ErC finding, considering it is a region appearing early in the Braak staging scheme and has been previously described as an epicenter of tau progression [1, 29–34]. For A36, which is typically affected in later stages of tau accumulation [33, 34], this is likely explained by the rank-order alignment of its connectivity profile with the MTL NFT gradient. Particularly, A36 shows its strongest functional connectivity with high-tau region A35, and weaker connectivity with lower-tau regions such as CA3 and the posterior subiculum. Furthermore, although the absolute functional connectivity of A36 with other high-tau regions such as the ErC and CA1 is modest, these regions nonetheless appear higher in the rank ordering of A36’s connectivity profile than most of the low-tau regions. In contrast, A35, an early tau region, shows relatively weak functional connectivity with CA1, one of the regions with high NFT burden, which may have reduced the alignment between its connectivity profile and the NFT distribution – even though its overall seed-based correlation remained on the higher end. A similar misalignment was observed for CA1 itself: despite its high NFT burden, CA1 showed a negative association between seed ROI connectivity and NFT burden. This was unexpected, as negative associations are more typically anticipated for low-tau regions, where pathology-free regions tend to be functionally connected to each other, as is seen for CA3 and DG [12]. For CA1, this likely reflects its prominent functional connectivity with CA3 and DG, consistent with the canonical trisynaptic circuit organization [56], and previously confirmed with resting-state fMRI [57]. As CA3 and DG are among the least tau-affected MTL regions, this resulted in a connectivity profile that shows limited correspondence with the regional NFT distribution. Importantly, as the seed-based analysis relies on Spearman correlations, we have to note that it captures the rank-order alignment between a seed's connectivity profile and the NFT distribution, rather than the absolute strength of connectivity or tau accumulation. This means that A36 emerges not necessarily because it is a strong functional hub, but because its connectivity profile best mirrors the ranked NFT distribution across the MTL. Furthermore, the relatively low functional connectivity of A36 may limit its absolute contribution to the rate of tau progression, as stronger connectivity has been associated with faster spread at the individual level [58]. Consequently, while our findings identify A36 as a contributor to MTL NFT distribution, we cannot define the precise absolute contribution of each region.

A key strength of the present study is the combination of high-resolution quantitative histopathology with ultra-high-field 7T fMRI, allowing us to relate post-mortem NFT burden to fine-grained functional connectivity within the MTL. The use of 3D-reconstructed histology provides anatomical precision not achievable with *in vivo* tau-PET, which is limited by partial volume effects and insufficient resolution to distinguish most individual MTL subregions [34–38]. However, several limitations must also be reported. First, functional connectivity was derived from a separate cohort of young healthy controls. Although the backbone of functional connectivity is relatively stable, it remains possible that connectivity patterns differ in young versus older and/or affected individuals, which could have influenced connectivity-tau associations [17, 59, 60]. Second, while unique, the post-mortem sample was relatively small (*n* = 25) and heterogeneous. However, since AD is rarely pure and frequently co-occurs with other pathologies [61], this heterogeneity is inherent to studying AD populations. Third, NFT pathology was assessed in a single hemisphere per individual, as the contralateral hemisphere was reserved for neuropathological diagnosis. As lateralization in tau burden is not uncommon, it cannot be excluded that this introduced some degree of bias in the regional estimates [62–64]. Fourth, the segmentation protocol was originally developed for *in vivo* MRI and applied here to both the post-mortem and *in vivo* fMRI dataset. Post-mortem data was therefore first labelled by an anatomist and subsequently adjusted according to the *in vivo* protocol. While this approach ensured high anatomical precision and comparability of ROIs across datasets, regions are not fully identical. Moreover, the resulting ROI boundaries are approximations of the true subfield borders, which may compromise anatomical validity – especially in smaller regions, where a small deviation from the true subfield boundary may have a larger impact [40–43]. Related to that, given the small size of the MTL subregions relative to fMRI resolution, some connectivity estimates may partly reflect spatial proximity rather than functional coupling between regions [49, 65].

The present findings provide novel evidence that tau distribution within the MTL is shaped by its intrinsic functional network organization, extending the concept of connectivity-based tau propagation to the granular, intra-MTL level. Our results suggest that tau patterns in the MTL best align with entorhinal and perirhinal connectivity across disease stages. Therefore, the stage at which tau pathology is still limited to the entorhinal and perirhinal cortices may define a critical intervention window, before further progression within the MTL and beyond, during which targeting tau propagation may be most effective. Future work should aim to replicate these findings in larger and more homogeneous cohorts and examine whether individual differences in MTL connectivity predict the rate or pattern of tau progression in AD.

## Supplementary Material

Supplementary Files

This is a list of supplementary files associated with this preprint. Click to download.

- SupplementarymaterialsJune29.docx

## Figures and Tables

**Figure 1:**
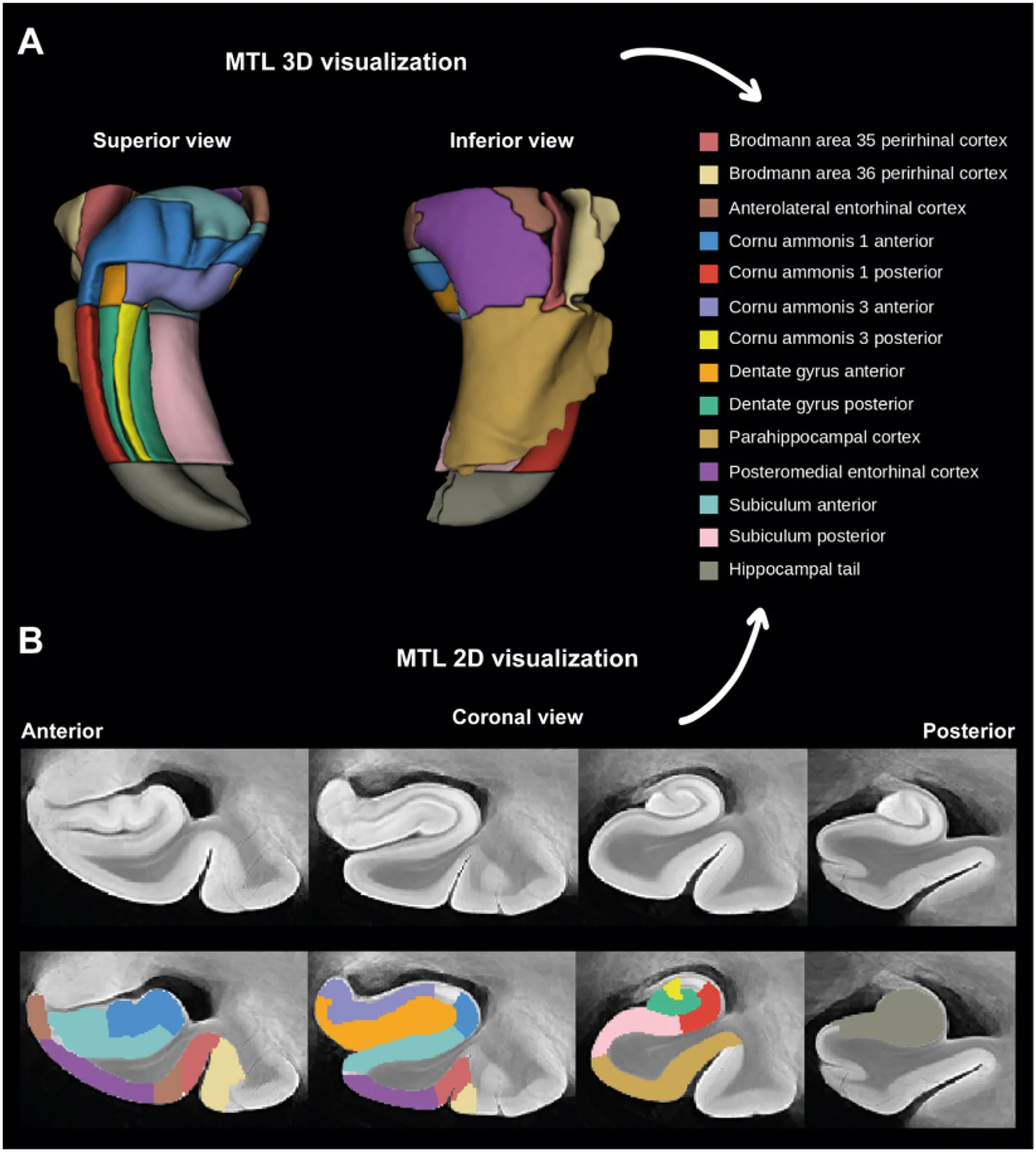
Atlas-based visualization of MTL ROIs. **(A)** Three-dimensional surface rendering and **(B)** coronal two-dimensional cross-sections showing the 14 MTL subregions delineated according to the Berron protocol [43]. Regions are color-coded as indicated in the legend (applicable to both A and B). CA2 was not included as a separate region of interest, hence the gap in the overlay of the images. MTL = medial temporal lobe; 2D = two-dimensional; 3D = three-dimensional.

**Figure 2:**
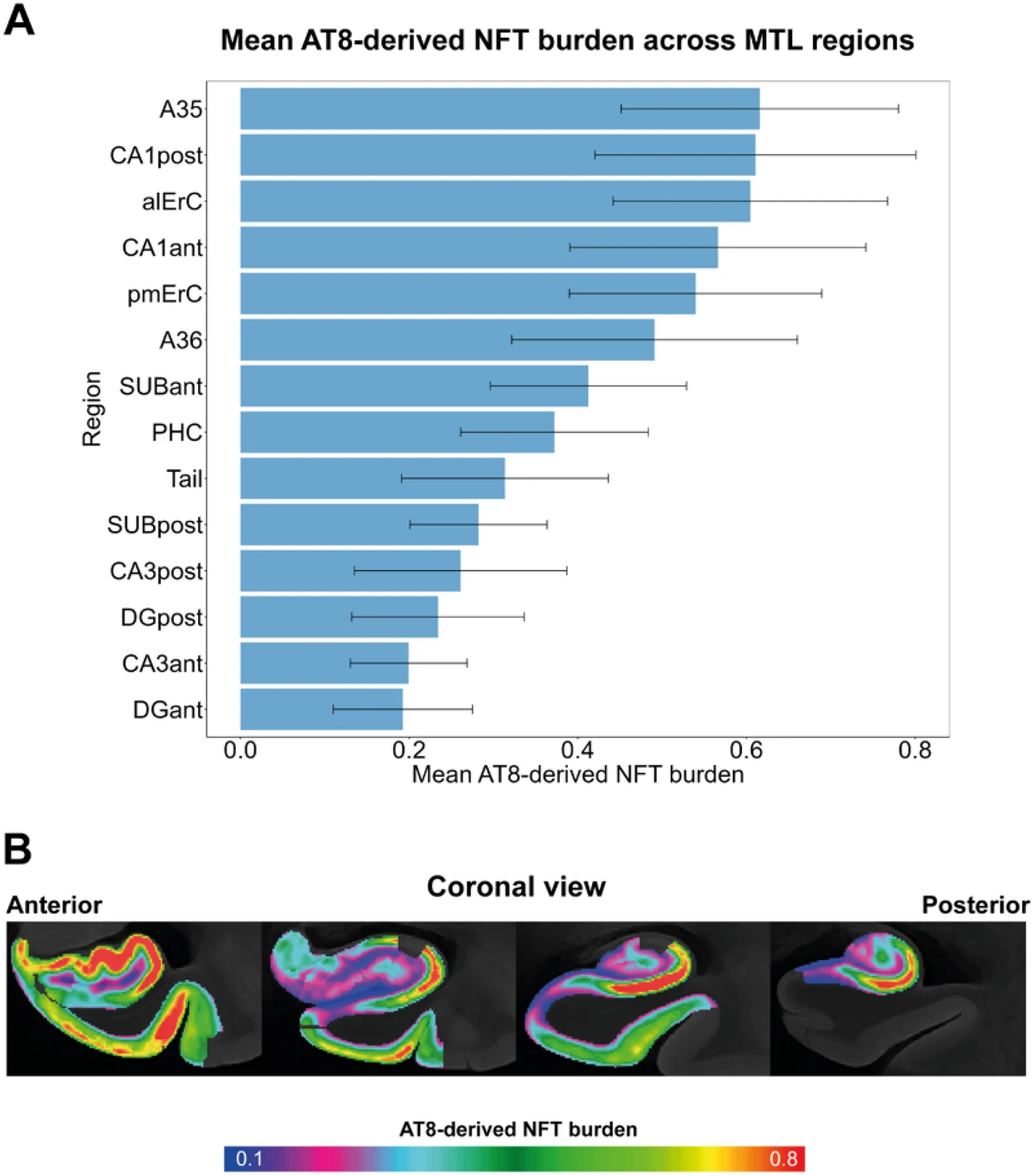
Mean AT8-derived NFT burden across MTL subregions. **(A**) Mean regional NFT burden per MTL subregion across 25 post-mortem donors, quantified from a single hemisphere per donor using AT8 immunostaining and a weakly supervised learning approach [33, 44]. For each region and donor, NFT burden was summarized as the mean of voxel values within the region, considering only voxels for which histology-derived measures were available. Regions are ranked from highest to lowest mean NFT load. Error bars represent 95% confidence intervals. (**B**) Voxel-wise average AT8 NFT burden visualized in template space across coronal sections from anterior to posterior. Color scale reflects NFT burden intensity (range 0.1–0.8). CA2 was not included as a separate region of interest, hence the gap in the overlay of the images. A35 = Brodmann area 35 (area 35 of the perirhinal cortex; transentorhinal cortex); A36 = Brodmann area 36 (area 36 of the perirhinal cortex); alErC = anterolateral entorhinal cortex; AT8 = anti-phosphorylated tau antibody; CA1ant = anterior cornu ammonis 1; CA1post = posterior cornu ammonis 1; CA3ant = anterior cornu ammonis 3; CA3post = posterior cornu ammonis 3; DGant = anterior dentate gyrus; DGpost = posterior dentate gyrus; MTL = medial temporal lobe; NFT = neurofibrillary tangle; PHC = parahippocampal cortex; pmErC = posteromedial entorhinal cortex; SUBant = anterior subiculum; SUBpost = posterior subiculum; Tail = hippocampal tail.

**Figure 3:**
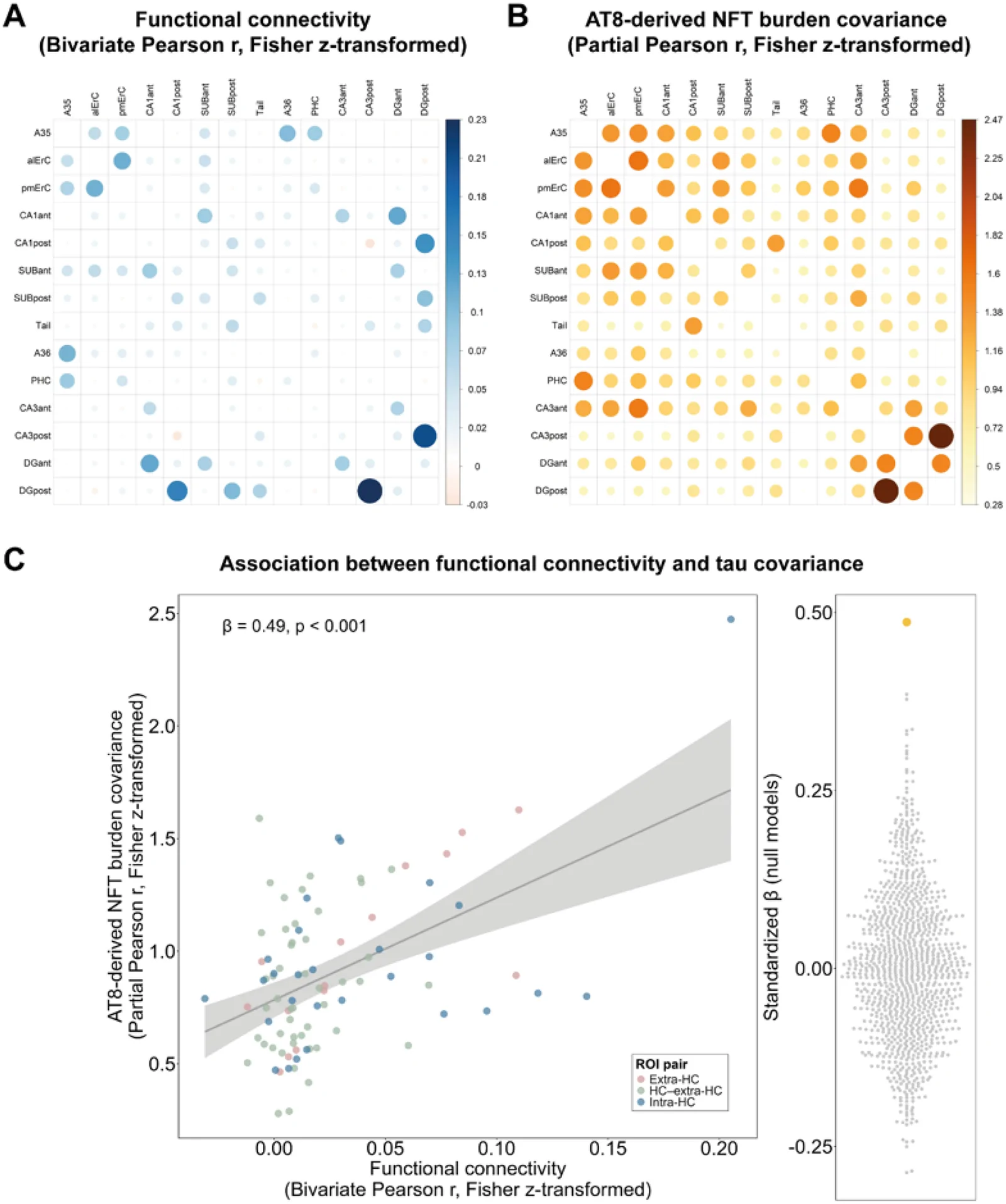
Association between within-MTL functional connectivity and post-mortem NFT covariance. **(A)**Pairwise ROI-to-ROI functional connectivity matrix (i.e., Fisher z-transformed bivariate Pearson correlations) derived from 30 young healthy controls who underwent high-resolution 7T resting-state fMRI. Circle size and color intensity reflect the magnitude of connectivity strength. **(B)** Pairwise NFT covariance matrix from 25 post-mortem donors, computed as partial Pearson correlations between regional AT8 NFT load across participants, adjusted for age and sex, and Fisher z-transformed. Circle size and color intensity reflect covariance magnitude. **(C)** Scatterplot showing the association between functional connectivity and NFT covariance across all ROI pairs. Each dot represents one ROI pair, and dots are color-coded by ROI pair type: intra-hippocampal (both ROIs are hippocampal subfields), extra-hippocampal (both ROIs are MTL cortical regions), or hippocampal–extra-hippocampal (one ROI of each type). The regression line with 95% confidence interval is shown. The standardized regression coefficient (*β*) is derived from linear regression with NFT covariance as the outcome and functional connectivity as the predictor; statistical significance was assessed against rewired functional connectivity null models, which are shown in the beeswarm plot (the observed *β* is indicated by the yellow dot). A35 = Brodmann area 35 (area 35 of the perirhinal cortex; transentorhinal cortex); A36 = Brodmann area 36 (area 36 of the perirhinal cortex); alErC = anterolateral entorhinal cortex; AT8 = anti-phosphorylated tau antibody; CA1ant = anterior cornu ammonis 1; CA1post = posterior cornu ammonis 1; CA3ant = anterior cornu ammonis 3; CA3post = posterior cornu ammonis 3; DGant = anterior dentate gyrus; DGpost = posterior dentate gyrus; HC = hippocampal; NFT = neurofibrillary tangle; PHC = parahippocampal cortex; pmErC = posteromedial entorhinal cortex; ROI = region of interest; SUBant = anterior subiculum; SUBpost = posterior subiculum; Tail = hippocampal tail.

**Figure 4:**
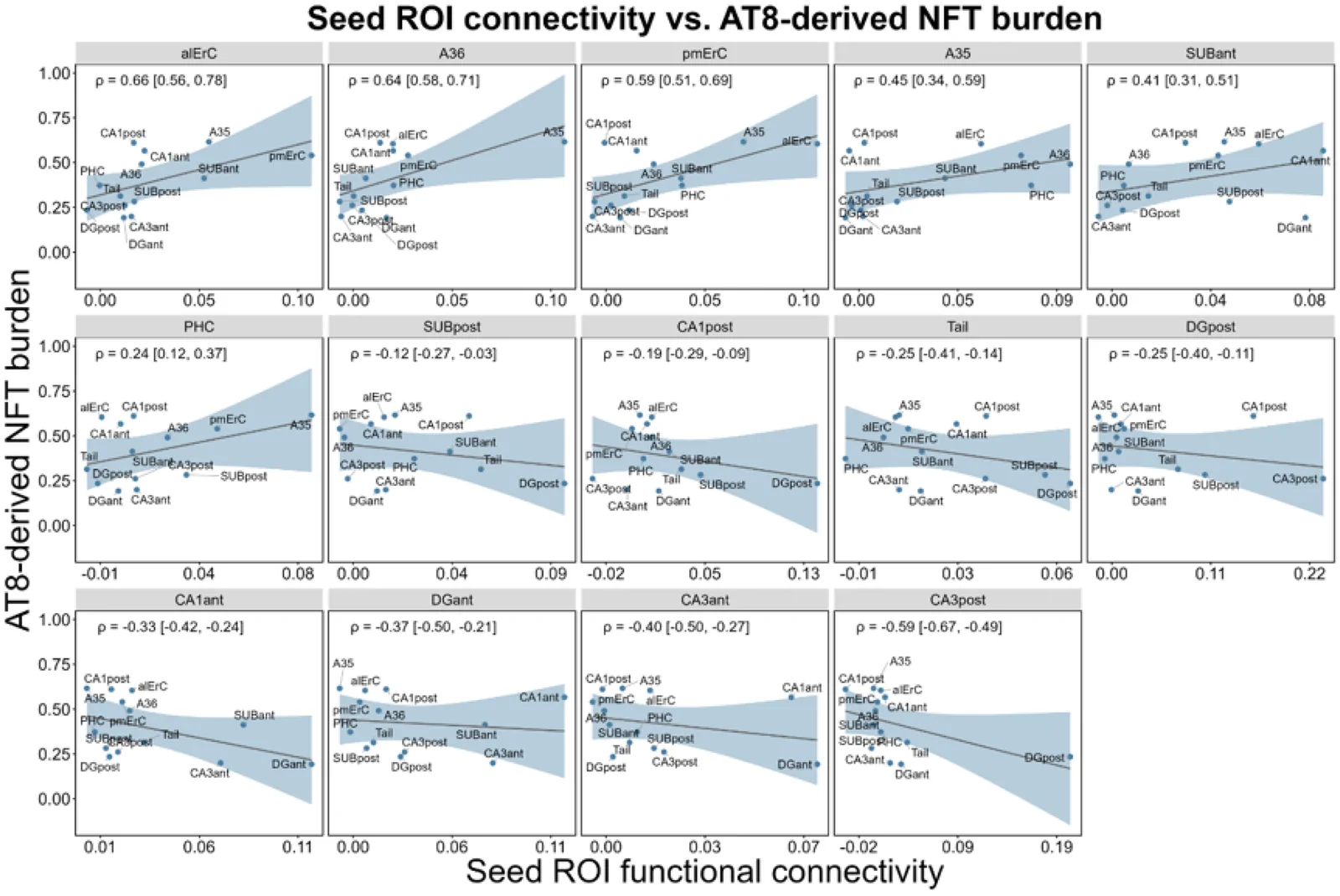
Association between seed ROI functional connectivity and post-mortem NFT load. For each of the 14 MTL subregions serving as a seed, the functional connectivity profile of that seed was correlated with the group-average AT8 NFT load in the remaining ROIs using Spearman rank correlations. A bootstrapping procedure (*n*= 1000 iterations, sampling subjects with replacement) was used to recompute the group-average NFT profile and seed-specific correlation. Each panel shows one seed ROI; points represent individual target ROIs. A linear regression line with 95% confidence interval is shown. The mean bootstrapped Spearman correlation with 95% confidence interval is displayed in the upper left corner of each panel. Panels are ordered from highest to lowest bootstrapped mean correlation. Pairwise differences between seed ROIs were tested using bootstrap-based tests with FDR correction. A35 = Brodmann area 35 (area 35 of the perirhinal cortex; transentorhinal cortex); A36 = Brodmann area 36 (area 36 of the perirhinal cortex); alErC = anterolateral entorhinal cortex; AT8 = anti-phosphorylated tau antibody; CA1ant = anterior cornu ammonis 1; CA1post = posterior cornu ammonis 1; CA3ant = anterior cornu ammonis 3; CA3post = posterior cornu ammonis 3; DGant = anterior dentate gyrus; DGpost = posterior dentate gyrus; NFT = neurofibrillary tangle; PHC = parahippocampal cortex; pmErC = posteromedial entorhinal cortex; ROI = region of interest; SUBant = anterior subiculum; SUBpost = posterior subiculum; Tail = hippocampal tail.

**Table 1:** Demographic and neuropathological characteristics of the post-mortem donor cohort. Continuous variables are displayed as mean (standard deviation). Categorical variables are displayed as *n* (%).

*n*	25
Age	78.60 (10.95)
Female	9 (36.0)
A (Aβ plaques)	
0	4 (16.0)
1	6 (24.0)
2	5 (20.0)
3	10 (40.0)
B (NFTs)	
0	1 (4.0)
1	10 (40.0)
2	10 (40.0)
3	4 (16.0)
C (NPs)	
0	8 (32.0)
1	5 (20.0)
2	4 (16.0)
3	8 (32.0)
Primary neuropathological diagnosis	
Low ADNC	8 (32.0)
Intermediate ADNC	6 (24.0)
High ADNC	3 (12.0)
Other*	8 (32.0)

Amyloid-β plaques,NFTs and NPs are presented according to ABC-score criteria (A = amyloid-β plaques using the Thal phase system; B = NFTs using the Braak staging system; C = NPs based on the CERAD criteria) [51]. *While donors were all on the AD continuum, other neuropathological diagnoses were also identified (i.e., Lewy body disease,Primary Age-Related Tauopathy [PART], or cerebrovascular disease). Aβ = amyloid-β; ADNC = Alzheimer's disease neuropathologic change; NFT = neurofibrillary tangle; NP = neuritic plaque.

## Data Availability

We obtained autopsy data from the OpenNeuro repository (https://openneuro.org/datasets/ds004767/versions/1.0.0).
